# Hyperpigmentation of hard palate induced by chloroquine therapy

**DOI:** 10.4317/jced.54387

**Published:** 2017-12-01

**Authors:** Bruno-Augusto-Benevenuto de Andrade, Nelson-Alejandro Padron-Alvarado, Edgar-Manuel Muñoz-Campos, Thayná-Melo-de Lima Morais, Ricardo Martinez-Pedraza

**Affiliations:** 1 DDS, PhD, Oral Pathology, Department of Oral Diagnosis and Pathology, School of Dentistry, Federal University of Rio de Janeiro (UFRJ), Rio de Janeiro, Brazil; 2 DDS, Oral Pathology, Universidad de Carabobo, School of Dentistry, Valencia, Venezuela; 3 DDS, Oral Surgery, University of Montemorelos, School of Dentistry, Montemorelos, NL, Mexico; 4 DDS, Oral Pathology Section, Department of Oral Diagnosis, Piracicaba Dental School, University of Campinas (UNICAMP), Piracicaba, São Paulo, Brazil; 5 DDS, Oral Pathology, Universidad Autónoma de Nuevo León, School of Dentistry, Monterrey, NL, Mexico

## Abstract

The antimalarials are one of the most commonly prescribed drugs for conditions such as lupus erythematosus and rheumatoid arthritis, and the side effects, though infrequent, are well known. The antimalarial agent chloroquine diphosphate usually causes pigmentary changes in the oral mucosa characterized by a bluish-grey to black discolorations mainly in the hard palate. Considering only the hard palate hyperpigmentation caused by chloroquine, to the best of our knowledge, only 13 cases have been reported in the English language literature. We described an additional case of palate hyperpigmentation related to the chronic use of chloroquine diphosphate in a 60-year-old Mexican woman. Although the diagnosis is usually made based on medication history and clinical presentation, a biopsy specimen may be helpful to confirm the diagnosis. Clinicians must be aware of these drugs and their adverse effects in order to make the correct diagnosis and decide on the optimal treatment for the condition.

** Key words:**Oral cavity, hard palate, hyperpigmentation, chloroquine, antimalarials.

## Introduction

Oral mucosal pigmentation is a common finding, usually associated with normal melanin deposition in dark-skinned people. A wide variety of lesions and conditions are associated with abnormal mucosal discoloration. Isolated and well-circumscribed oral pigmented lesions are usually diagnosed as melanotic macule, melanocytic nevus or amalgam tattoo, or more uncommonly as an initial sign of melanoma. Diffuse or multifocal mucosal hyperpigmentation may be a sign of systemic disease such as Addison’s disease, Peutz–Jeghers syndrome, melanoplakia and human immunodeficiency virus (HIV) infection, or a side effect of drug therapy (1-5).

Drugs associated with abnormal oral pigmentation include tetracycline, zidovudine, anti-inflammatory drugs and antimalarial agents, such as quinacrine hydrochloride, chloroquine, hydroxychloroquine, and amodiaquine (6). In addition to treating malaria, these medications are used for management of systemic and discoid lupus erythematosus and rheumatoid arthritis (7-18). Clinicians will most likely encounter patients taking these medications and should, therefore, be familiar with this potential oral side effect (1). Early diagnosis of oral pigmentation by antimalarials may be of great relevance, since it might be an early sign of ocular involvement, and therefore it may be helpful to prevent further complications of antimalarial therapy for the patient (1). We describe an additional case of hard palate hyperpigmentation related to the chronic use of chloroquine diphosphate for rheumatoid arthritis treatment.

## Case Report

**Figure 1:**
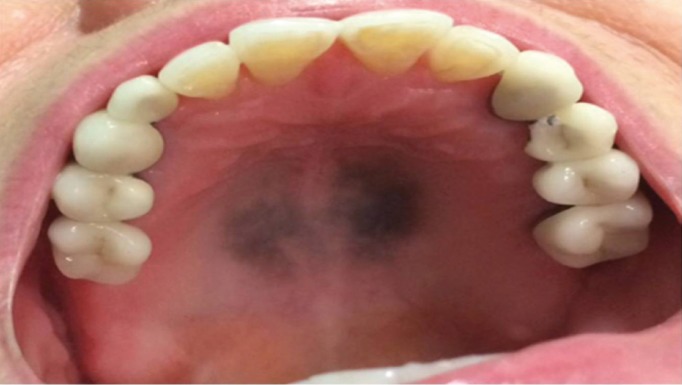
Clinical aspect of hyperpigmentation of hard palate induced by chloroquine therapy showing a blue-gray pigmented diffuse lesion with irregular borders.

A 60-year-old Mexican woman was referred for evaluation of a diffuse blue-gray pigmentation of the hard palate lasting six months. Her medical history revealed that she had been undergoing treatment with chloroquine diphosphate (150mg/day) for rheumatoid arthritis for 1 year. Clinical examination showed a 4 cm blue-gray pigmented diffuse lesion with irregular borders on the hard palate (Fig. 1). The pigmented area did not blanch with pressure. On extra-oral examination, pigmentation was not seen in the skin or in the ocular conjunctiva. Differential diagnosis included drug-induced hyperpigmentation, Addison’s disease, vitamin B12 deficiency, and melanoma. The history of long-term chloroquine use, led to the clinical working diagnosis of drug-induced oral pigmentation caused by chloroquine diphosphate. To confirm this, an incisional biopsy was taken from the hard palate mucosa and sent for histopathological examination.

**Figure 2:**
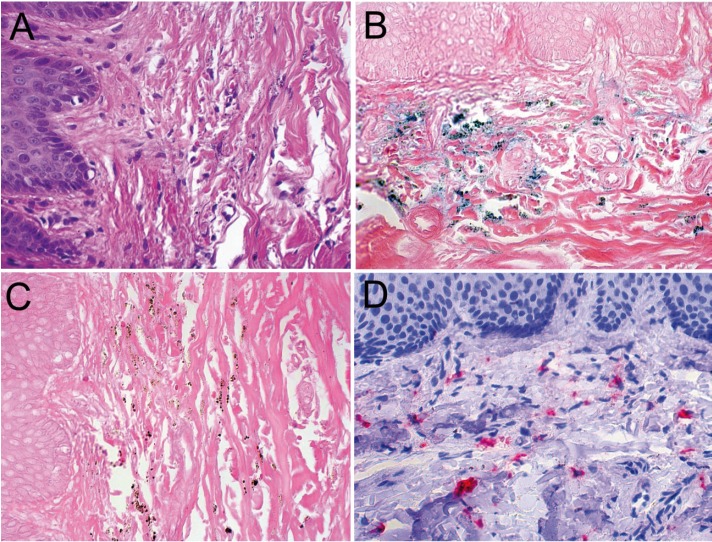
Histological aspects of hyperpigmentation of hard palate induced by chloroquine therapy. A – Subepithelial deposition of granular pigment mainly located between collagen fibers and within fibroblasts and macrophages (HE, 400X). B – Staining with Perls’ confirmed that the pigment present in the lesion is constituted by hemosiderin (Perls’ stain, 400X). C – The pigment deposits were also positive for Fontana-Masson stain (400X). D – Immunohistochemical aspects of hyperpigmentation of hard palate induced by chloroquine therapy. Presence of intracellular dark-brown pigment in the macrophages with CD68 (Permanet red, 400X).

Microscopical evaluation showed a subepithelial deposition of granular pigment mainly located between collagen fibers and within fibroblasts and macrophages. Staining with Perls’ confirmed that the pigment was hemosiderin. Fontana-Masson stain was also positive confirming the presence of melanin (Fig. 2). Immunohistochemistry with CD68 (dilution 1:400, clone PGM-1, Dako, Carpinteria, CA, USA) highlighted macrophages containing intracellular pigment (Fig. 2). These histopathological findings and the clinical appearance of the lesion confirmed the diagnosis of drug-induced oral pigmentation caused by chloroquine diphosphate. The drug was discontinued and the patient was referred for ophthalmologic evaluation that showed no signs of retinopathy.

## Discussion

Oral mucosal pigmentation can be result of a wide variety of lesions and conditions. A systematic evaluation, including a complete and accurate patient history and a thorough clinical examination, is essential for the appropriate differential diagnosis (2). Brown, black, or gray discoloration is most often caused by an accumulation of melanin, hemosiderin, or foreign body material, whereas red, blue, or purple color changes suggest a vascular process (2). Multifocal or diffuse distribution of pigmentation suggests a systemic cause, such as a metabolic disorder or drug toxicity. Antimalarial agents, such as chloroquine diphosphate and hydroxychloroquine sulfate, are administered for treatment of several dermatologic and rheumatologic disorders, and they are known to cause hyperpigmentation of the oral mucosa (9,15). Systemic administration of these drugs for a prolonged period is responsible for the appearance of multifocal hyperpigmentation, which is reversible once the medication is discontinued. Oral pigmentation secondary to drug therapy can be attributed to the stimulation of melanin production by melanocytes and/or the deposition of hemosiderin in the tissues (1,2,5).

Lippard and Kauer first described pigmentation of palatal mucosa resulting from antimalarial medication in 1945 (10). Since then, it has been reported by others (1,5,10,11). In most cases, only the hard palate is involved, forming a sharp line of demarcation at the junction of the hard and soft palates (2). An explanation for sparing the soft palate has not been offered. Involvement of gingiva and labial or buccal mucosa has also been reported (4). Considering only the hard palate hyperpigmentation caused by chloroquine, to the best of our knowledge, only 13 cases have been reported in the English language literature ( Table 1) (1,3-5,12,16-18). Skin pigmentation can also be observed in patients undergoing treatment with chloroquine diphosphate (2) as well as reversible graying of the scalp hair, beard, eyelashes, and eyebrows (14). Pigmentary changes in the oral mucosa can also be associated with other medicaments including tranquilizers (chlorpromazine), chemotherapeutics (doxorubicin, busulfan, and cyclophosphamide), anti-retroviral agents (zidovudine, AZT), antifungal agents ketoconazole), antibiotics (minocycline), and laxatives (phenolphthalein) (13). In the current case, the hyperpigmentation was localized only in the hard patale without cutaneous involvement.

It is important to note that several systemic disorders can promote oral and cutaneous pigmentation and this should be further explored through the medical history. Systemic causes that need to be considered include adrenal insufficiency, Peutz- Jeghers syndrome, hemochromatosis, polyostotic fibrous dysplasia, hyperparathyroidism, and neurofibromatosis (2). Abnormal pigmentation is relatively common in individuals infected with the human immunodeficiency virus (HIV). In some HIV-related cases, pigmentation has been associated with drug therapy or adrenal insufficiency; however, in many cases the cause cannot be identified (15).

**Table 1:**
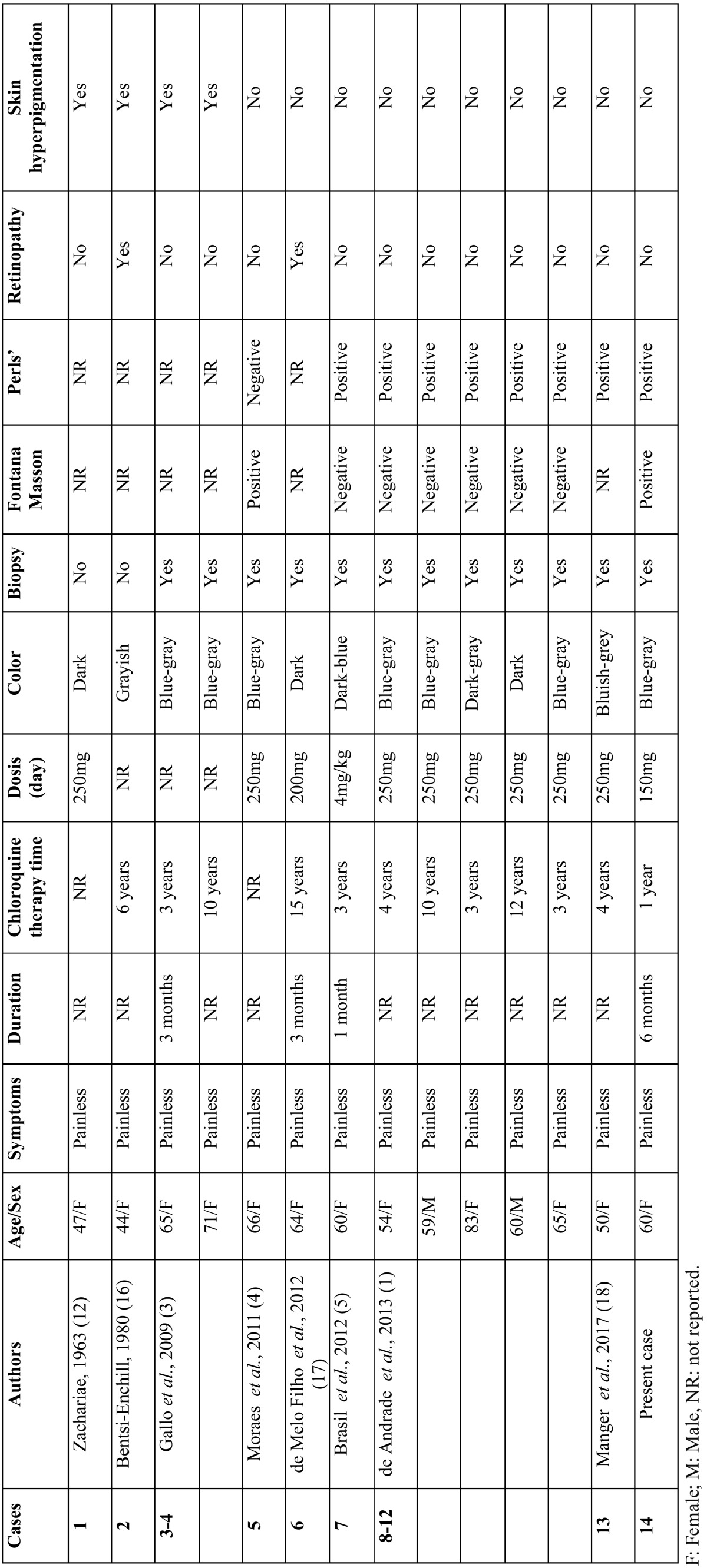
Clinical and microscopical features of 12 cases of hard palate hyperpigmentation induced by chronic chloroquine therapy reported in the English-language literature including the present case.

The greatest significance of chloroquine-induced hyperpigmentation is the possibility that it may be a marker for a more serious side effect. Irreversible retinopathy which in some cases leads to blindness, is recognized as a potential complication of antimalarial drug therapy and it has been suggested that abnormal skin and mucosal pigmentation may be an indication of ocular involvement (2,3). Based on this potentially severe complication, periodic evaluation is necessary for patients being treated with ongoing antimalarial therapy. Our patient was evaluated by an ophthalmologist showing no signs of retinopathy.

The diagnosis of drug-induced hyperpigmentation is often made based on medication history and clinical presentation. In cases which clinical features are atypical or a complete medication history is not available, a biopsy should be performed to establish the diagnosis (2). Biopsy is particularly important to rule out melanoma, which may initially present as hyperpigmentation of otherwise normal appearing mucosa (2,5).

In chloroquine-induced hyperpigmentation, biopsy specimens of involved mucosa may exhibit gross subepithelial pigmentation (1). Hematoxylin-eosin stained sections demonstrate deposits of granular pigmentation extracellular or within fibroblasts and macrophages scattered throughout the lamina propria. Histochemical stains have been used in an attempt to identify the nature of the pigmentation; however, results have been inconsistent. Authors have identified melanin, hemosiderin, or both (1,5). In our case we confirmed presence of hemosiderin and melanin as the pigment was positive with wither Perls’ and Fontana Masson stainings.

For this type of oral pigmentation, no treatment is required. The management involves, if possible, discontinuing the medication or decreasing the dosage, and it has been recommended that these patients also be referred to an ophthalmologist (1,3).

In summary, diffuse oral pigmentation can be a sign of drug side effect and should be included as part of the clinical differential diagnosis of hyperpigmentation of the oral mucosa. Antimalarial agents such as chloroquine are among the drugs more commonly associated with this mucosal alteration. Although the diagnosis is usually made based on medication history and clinical presentation, a biopsy specimen may be helpful to confirm the diagnosis. The management involves discontinuing or decreasing use of the drug and referral for ophthalmologic examination.
